# Diagnostic Dilemmas in Carpal Tunnel Syndrome and Cervical Spine Disorders: A Comprehensive Review

**DOI:** 10.3390/diagnostics15020122

**Published:** 2025-01-07

**Authors:** Yuki Hara, Yuichi Yoshii

**Affiliations:** 1 Department of Orthopedic Surgery, National Center of Neurology and Psychiatry, Kodaira 187-8551, Tokyo, Japan; 2 Department of Orthopedic Surgery, Tokyo Medical University Ibaraki Medical Center, Ami 300-0395, Ibaraki, Japan

**Keywords:** carpal tunnel syndrome, cervical radiculopathy, cervical spondylotic myelopathy, differentiation diagnosis

## Abstract

Carpal tunnel syndrome (CTS) and cervical spondylosis (CS) are both common diseases, yet differentiation between the two is sometimes necessary. However, there are few evidence-based reviews on the differentiation of these conditions. This review examined the literature on the diagnosis of CTS and CS, focusing on how to distinguish between them. The analysis is divided into four categories: clinical symptoms, physical examination, diagnostic imaging, and electrodiagnosis. A total of 281 studies are reviewed, revealing a major issue: the inclusion criteria for defining each disease varies widely across studies. Understanding this limitation, the conclusion drawn is that no single clinical symptom, test, or imaging evaluation can be deemed uniquely reliable for diagnosing CTS or CS. Therefore, it is essential to apply the most up-to-date knowledge, conduct thorough examinations, and perform necessary tests for each patient to achieve a confident and accurate diagnosis.

## 1. Introduction

Differentiating between carpal tunnel syndrome (CTS) and cervical spine disorders such as cervical radiculopathy (CR) or cervical spondylotic myelopathy (CSM) is often a clinical challenge due to overlapping symptoms and the absence of globally standardized diagnostic criteria. Carpal tunnel syndrome is a condition that occurs when the median nerve is compressed within the carpal tunnel in the wrist. Cervical radiculopathy is a compressive nerve root disorder in the cervical spine, while cervical spondylotic myelopathy is a condition where the spinal cord is compressed in the cervical spine. When these conditions occur at the C5–6 level, symptoms of the C6 nerve root and C6 myotome dysfunction arise, which involve the median nerve distribution, making it necessary to differentiate from carpal tunnel syndrome. Symptoms such as pain, numbness, and weakness in the upper extremities can result from either CTS or cervical spine disorders, making their accurate diagnosis imperative for effective treatment [1,2,3,4,5]. A major issue contributing to this diagnostic challenge is the lack of a universally accepted gold standard for diagnosing either condition, which adds to the variability in clinical practices and research methodologies [6].

For CTS, even though it is one of the most studied peripheral neuropathies [7,8,9,10,11], there is no consensus on diagnostic criteria, as evidenced by the vast number of published papers each year with heterogeneous inclusion criteria. Similarly, CR and CSM also suffer from variability in diagnostic approaches. A systematic review of intervention studies for cervical radiculopathy highlighted significant inconsistencies in patient selection criteria across trials, further complicating efforts to establish a uniform diagnostic approach [12].

This lack of standardization in diagnostic criteria not only affects clinical decision-making but also impacts the interpretation and generalization of research findings [13,14,15,16]. The unique advantages of the various testing methods for CTS diagnosis have been widely discussed [16]. Accurate differentiation is particularly critical as misdiagnosis can lead to inappropriate treatments, which may exacerbate symptoms or delay proper intervention. For instance, CTS may be mistaken for cervical spondylosis (CS), leading to unnecessary spinal interventions, or vice versa, potentially delaying necessary peripheral nerve decompression. Furthermore, conditions like diabetic polyneuropathy can mimic or coexist with CTS and cervical spondylosis, adding another layer of complexity to the diagnostic process.

This comprehensive review focuses specifically on the differentiation between CTS and CS, two conditions with distinct pathophysiologies but overlapping clinical presentations. The aim is to review and synthesize the existing literature on the diagnostic methods for each condition, highlighting key studies that address their differentiation. By analyzing the strengths and limitations of current diagnostic approaches, this study seeks to provide insights into improving clinical practices and fostering the development of standardized criteria. Ultimately, accurate and reliable differentiation between CTS and cervical spondylosis is essential to guide patients toward the most appropriate treatment, reduce the burden of misdiagnosis, and improve overall outcomes.

## 2. Materials and Methods

A search of the PubMed MeSH database was conducted to identify literature from the past 10 years written in English that describes the conditions of interest. The following search terms were used: “Carpal Tunnel Syndrome/diagnosis” [MAJR] OR “Carpal Tunnel Syndrome/diagnostic imaging” [MAJR] with filters for the past 10 years, full-text availability, clinical trials, meta-analyses, randomized controlled trials (RCTs), reviews, and systematic reviews, yielding 154 results; “Spinal Cord Compression/diagnosis” [MAJR] OR “Spinal Cord Compression/diagnostic imaging” [MAJR] with the same filters, yielding 52 results; and “Radiculopathy/diagnosis” [MAJR] OR “Radiculopathy/diagnostic imaging” [MAJR] with identical filters, yielding 75 results.

A total of 281 papers were reviewed. However, due to the limited number of studies specifically addressing the differentiation between CTS and cervical spondylosis, a broader review of the diagnostic approaches for CTS, cervical radiculopathy (CR), and cervical spondylotic myelopathy (CSM) was conducted. Among the reviewed articles, only five studies, including both original research and review articles, specifically focused on differentiating between carpal tunnel syndrome and cervical spondylosis [3,4,5,6,7]. The studies were categorized based on their approach to the differential diagnosis between carpal tunnel syndrome and cervical spondylosis, focusing on clinical symptoms, imaging examinations, and electrodiagnostic methods. These five key studies provide valuable insights into identifying distinguishing features between these conditions, addressing a critical gap in the current diagnostic framework.

## 3. Results

### 3.1. Studies on the Differences in Clinical Symptoms Between CTS and CS

Clinical symptoms and examination findings are positioned as the initial screening in the diagnostic process. In the differential diagnosis of diseases with similar symptoms, the task involves extracting findings characteristic of each disease from among the many observations obtained. A review was conducted on studies comparing differences among similar diseases as well as studies focusing on the clinical features of each disease. The findings were extracted from studies on the clinical symptoms and physical examination of CTS and CS that are useful for distinguishing between the two diseases. There are five studies that primarily focus on differentiating CTS and CS based on clinical symptoms.

Chow et al. conducted a retrospective comparison of 44 patients who underwent surgery for carpal tunnel syndrome and 41 patients who underwent spinal surgery for cervical spondylosis, reporting differences in preoperative symptoms [17]. In carpal tunnel syndrome, 84% of patients experienced nocturnal paresthesia, 82% had paresthesia worsened by hand activity, and 64% reported hand pain. In contrast, these symptoms were significantly less frequent in cervical spondylosis, occurring in 10%, 7%, and 10% of patients, respectively. Neck pain was present in 76% of cervical spondylosis cases but only in 14% of carpal tunnel syndrome cases. Additionally, lower extremity symptoms were observed in 44% of cervical spondylosis patients compared to just 9% of those with carpal tunnel syndrome.

Lo et al. reported on the clinical and electrodiagnostic characteristics of CTS, CR, and the coexistence of these conditions, known as double crush syndrome (DCS), as causes of unilateral upper extremity neurological symptoms [18]. They retrospectively analyzed the medical records and electrodiagnostic reports of 866 patients referred to a tertiary care center with suspected CTS or CR. After excluding 101 patients with confounding comorbidities, they categorized the cases as follows: CTS alone: 151 cases (20%), CR alone: 362 cases (47%), DCS: 198 cases (26%), and symptom-only patients: 54 cases (7%). An analysis of the clinical symptoms revealed that CR-alone cases showed higher rates of neck pain, upper back pain, and wrist or hand weakness compared to the other groups. In nerve conduction studies, statistically significant differences were observed between symptom-only patients and those with CTS or DCS alone; however, no differences were noted between CTS and DCS cases. The study highlighted that a large proportion of patients referred for electrodiagnostic evaluation had CR and pointed to a high coexistence rate of CTS and CR, emphasizing the importance of accurate diagnosis.

Tampin et al. studied patients with electrodiagnostically confirmed CTS (*n* = 103) and CR (*n* = 23), using healthy participants as a control group (*n* = 39) [19]. Symptoms and sensory profiles were evaluated using quantitative sensory testing (QST) and the self-reported neuropathic pain questionnaire painDETECT. Both patient groups were characterized by the loss of thermal sensation and mechanical detection functions at the main pain site and within the affected dermatome compared to healthy reference data (*p* < 0.001). No significant differences in pain or detection thresholds were observed between the CTS and CR groups. However, CR patients showed significantly reduced vibration sensation at the main pain site (*p* < 0.001) and decreased pressure pain sensitivity in the dermatome (*p* < 0.001) compared to CTS patients. Additionally, CR patients reported higher pain intensity (*p* = 0.008), more severe pain attacks (*p* = 0.009), and pain induced by light pressure (*p* = 0.002).

Marco et al. reported that when diagnosing cervical spondylotic radiculopathy based on the sensory disturbance range, there are cases where sensory disturbances extend beyond the affected nerve root, presenting overlapping symptoms across various nerve distribution areas [20]. Pain drawing diagrams are commonly used in the clinical evaluation of cervical radiculopathy patients. The concordance between the clinical interpretation of pain diagrams and MRI findings in identifying the affected level of cervical radiculopathy was generally low, with an average agreement rate of 35.7% and kappa values ranging from −0.007 to 0.139. The inter-rater agreement among clinicians was considered “fair” to “moderate”, with kappa values ranging from 0.212 to 0.446. Spatial distribution analysis showed that the pain distribution was widespread and significantly overlapped between patients with different levels of radiculopathy.

The most evidence-based review on the diagnosis of CTS to date is likely the 2022 review by Padua L, published in The Lancet [21]. It is recommended to avoid relying on a single clinical parameter when making a diagnosis. While clinical history and physical examination are essential for screening, their diagnostic accuracy varies. Although the specificity of various clinical examination items and patient-reported questionnaires or scoring systems is high, their sensitivity is relatively low.

### 3.2. Studies on the Differences in Provocation Tests Between CTS and CS

Verhagen et al. and Shen et al. reported a systematic review evaluating the diagnostic criteria for a positive upper limb tension test (ULTT) [22,23]. Four studies focused on diagnosing CR, four on CTS, and one on both conditions. However, the risk of bias varied across these studies, ranging from two to six out of six items. Eight studies specifically reported on ULTT1 (median nerve). While all studies clearly described the test procedures and positive diagnostic criteria, differences in the sequence of movements and diagnostic thresholds were noted among studies. The authors concluded that greater standardization is necessary to enhance consistency.

In 2022, Núñez de Arenas-Arroyo et al. performed a meta-analysis of 37 studies on the accuracy of the most common provocation tests for diagnosing carpal tunnel syndrome [24]. The pooled diagnostic odds ratios (dOR) for CTS screening were as follows: 15.84 (95% CI: 3.78, 66.38) for the Durkan test (DT), 128.63 (95% CI: 40.64, 407.12) for the hand elevation test (HET), 7.23 (95% CI: 4.06, 12.86) for the Phalen test (PT), 5.31 (95% CI: 3.49, 8.09) for the Tinel test (TT), and 1.78 (95% CI: 0.61, 5.19) for the Upper Limb Neurodynamic Test 1 (ULNT1). Therefore, the HET demonstrated the best clinical performance for detecting CTS and should be considered as the first screening test during physical examinations. The most common tests, including the DT, PT, and TT, also showed good accuracy for CTS screening.

However, in 2023, Dabbagh A and colleagues analyzed studies on provocative tests such as the Phalen test and Tinel’s sign [25]. While the Phalen test is generally considered to have moderate sensitivity and specificity and the Tinel’s sign is known for low sensitivity but high specificity, the studies revealed unclear and high risks of bias. As a result, they did not support the use of standalone provocative tests for the diagnosis of carpal tunnel syndrome (CTS). They concluded that clinicians should consider combining non-invasive clinical diagnostic tests as the initial approach to diagnosing CTS. These findings suggest that commonly performed provocative tests for suspected CTS differ in sensitivity and specificity [26].

Thoomes’ review summarizes the evidence regarding the diagnostic performance of physical examination tests conducted for the diagnosis of cervical radiculopathy [27]. Only the Spurling test was evaluated in multiple studies, showing a high specificity ranging from 0.89 to 1.00 (95% confidence interval [CI]: 0.59–1.00), while the sensitivity varied from 0.38 to 0.97 (95% CI: 0.21–0.99). No studies were found that assessed the diagnostic accuracy of widely used neurological tests, such as key muscle strength, tendon reflexes, or sensory impairments. The conclusion was that when consistent with the patient’s history, clinicians may increase the likelihood of CR by combining the Spurling test, axial traction, and the Arm Squeeze test. In another systematic review, it has been suggested that when consistent with the history and other physical findings, a positive Spurling test, traction/neck distraction, and Valsalva’s maneuver might be indicative of a cervical radiculopathy, while a negative ULTT might be used to rule it out [28]. However, there was limited evidence for the accuracy of physical examination tests for the diagnosis of cervical radiculopathy [27].

### 3.3. Studies on the Differences in Diagnostic Imaging Between CTS and CS

Imaging studies are performed as a means to demonstrate the presence of the lesion itself. In the differential diagnosis of CTS and CS, it is crucial to confirm through imaging whether a lesion explaining the symptoms exists in the carpal tunnel involving the median nerve or around the cervical spine, in conjunction with clinical findings suggestive of these conditions [29,30,31]. The latest studies on this topic are listed below. No studies comparing imaging findings between CTS and CS exist; instead, a review was conducted on studies focusing on the imaging diagnostics of each condition individually.

#### 3.3.1. Magnetic Resonance Imaging (MRI)

The literature review was conducted on how various lesions are identified using MRI. Both CTS and CS are classified as compressive neuropathies and currently diffusion tensor imaging (DTI) is considered the most sensitive method for detecting the affected nerves under compression. There were three reviews on diffusion tensor imaging (DTI) related to CTS [32,33,34]. Magnetic resonance DTI can detect microstructural changes in peripheral nerves. In the diagnosis of CTS, the apparent diffusion coefficient (ADC) of the median nerve has been reported to be a sensitive indicator for quantifying the direction of water molecule diffusion.

Evans AG and colleagues conducted a meta-analysis focusing on studies that used the ADC [32]. A total of 22 studies met the inclusion criteria, comprising 592 CTS patients and 414 controls. The analysis showed that the ADC of the median nerve was lower in CTS patients compared to controls at the levels of the hamate and pisiform bones. However, they noted that the cutoff value for ADC in CTS diagnosis varies depending on the position of the imaging slices and the coils used, posing a challenge due to discrepancies across different institutions.

Rojoa D, et al. reported a systematic review on diffusion tensor imaging examined the normal fractional anisotropy (FA) and mean diffusivity (MD) of the median nerve [33]. The review included 32 studies involving 2643 wrists, 1575 asymptomatic adults, and 1068 CTS patients. The normal FA was 0.58 (95% CI: 0.56, 0.59), and the normal MD was 1.138 × 10^−3^ mm^2^/s (95% CI: 1.101, 1.174). CTS patients demonstrated significantly lower FA (mean difference: 0.12 [95% CI: 0.09, 0.16]) and significantly higher MD (mean difference: 0.16 × 10^−3^ mm^2^/s [95% CI: 0.05, 0.27]) compared to controls. Furthermore, it was shown that diffusion in the median nerve became more isotropic in the CTS patients.

Liu C and colleagues conducted a meta-analysis to determine the optimal anatomical slice location for evaluating the median nerve using MRI in carpal tunnel syndrome [34]. They reported that fractional anisotropy (FA) was most suitable for diagnosis at the level of the pisiform bone, while the apparent diffusion coefficient (ADC) was more appropriate at the levels of both the pisiform and hamate bones.

In cervical spondylosis, DTI is effective for detecting changes in neural tissue at the site of compression on MRI images [35]. However, it is considered useful to combine DTI with other modalities, such as CT, to better capture the compression of nerve roots on imaging.

Meacock J and colleagues conducted a systematic review to identify a standardized method for evaluating cervical nerve root compression in cervical radiculopathy [36]. They searched the Ovid Medline, Embase (1947 to present), Cinahl, Web of Science, Cochrane Library, ISRCTN, and WHO International Clinical Trials databases for reports on cervical foraminal stenosis published before 1 February 2020. Most studies utilized multiple imaging modalities, with standard axial and sagittal imaging being the most commonly employed techniques. According to the grading themes derived from this systematic review, the most established systems for evaluating cervical foraminal stenosis were described by Kim et al. [31] and Park et al. [37]. The review concluded that oblique fine-slice images obtained from three-dimensional MRI datasets provide greater consistency, enhance clinical correlation, and have the potential to improve surgical decision-making and outcomes.

#### 3.3.2. Ultrasound (US)

Over the past two decades, high-resolution ultrasound has increasingly played a key role in the assessment of carpal tunnel syndrome [21,38]. Meta-analyses have evaluated the diagnostic accuracy of this approach, with cutoff values for the cross-sectional area at the carpal tunnel inlet ranging from 9.0 mm^2^ to 12.6 mm^2^ [21,39,40,41]. Additionally, there was an association between the size of the median nerve at the carpal tunnel inlet and the electrophysiological severity, with the mean cross-sectional area being 11.64 mm^2^ and increasing to 16.90 mm^2^ in the most severe cases based on nerve conduction test assessments [39,40,41].

**Figure 1:**
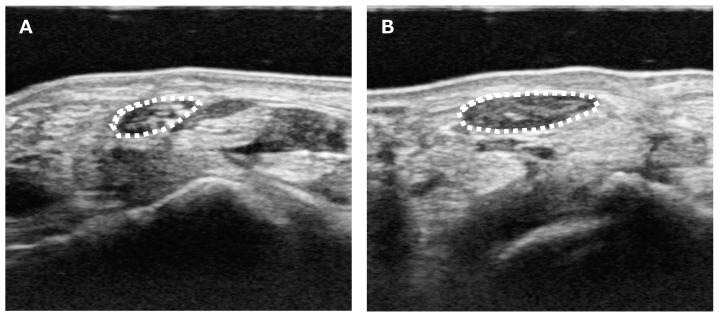
Ultrasound evaluations of median nerve cross-sectional area in unilateral CTS patients. (**A**) Unaffected side. (**B**) Affected side. Median nerve is enlarged in the affected side [43].

However, Chen et al. compared the CSA between diabetic (DM) patients and non-diabetic patients, noting that while CSA is a useful finding for diagnosing carpal tunnel syndrome in DM patients, caution is needed as CSA may be smaller in the presence of diabetic neuropathy [42]. In other study, Ikumi et al. investigated the relationships between the median nerve cross-sectional area and physical characteristics in patients with unilateral symptomatic CTS [43]. It has been suggested that the relationship between median nerve cross-sectional area (CSA) and body mass index (BMI) in CTS is significant until symptom onset but may be masked by edema and pseudoneuroma after its onset. A higher BMI is associated with a larger CSA of the median nerve, which may be a risk factor for the development of CTS (Figure 1).

In recent years, ultrasound diagnosis has gained attention as an alternative method for evaluating cervical nerve root injury. In a study by Kim et al., high-resolution ultrasound was used to measure the CSA of the cervical spinal nerve roots in patients with cervical radiculopathy, comparing the CSA of the affected and unaffected sides [44]. The study focused on patients diagnosed with unilateral cervical radiculopathy. The selected nerve roots were imaged ultrasonographically at the closest position to the transverse process, slightly distally from that point. The study involved twenty-four patients (nine women, average age 53.7 years). CSA was measured using ultrasound in five pairs of C5 nerve roots, twelve pairs of C6 nerve roots, and seven pairs of C7 nerve roots. The mean CSA of the affected and unaffected sides was 9.74 ± 1.95 mm and 9.47 ± 1.95 mm, respectively (*p* = 0.019). Spearman’s rank correlation test revealed a positive correlation between the CSA of the affected nerve root and the duration of symptoms (ρ22 = 0.467, *p* = 0.021). The increased CSA of the affected nerve root compared to the unaffected side may be a useful, inexpensive, and simple supplementary clue for diagnosing cervical radiculopathy.

### 3.4. Studies on the Differences in Electrodiagnosis Between CTS and CS

For physicians specializing in neurology, electrodiagnostic testing represents the assessment of nerve function itself. While the importance of electrodiagnostic testing in differentiating CTS from CS is well known, not all physicians involved in the treatment of CTS and CS are proficient in electrodiagnosis. A review of the latest studies was conducted, emphasizing the significance of electrodiagnosis once again.

**Figure 2:**
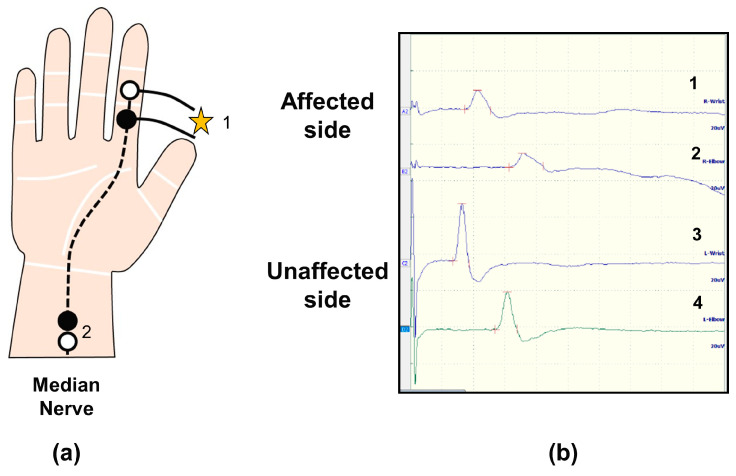
Typical findings of sensory nerve conduction study in CTS patients. (**a**) Electrode settings. 1: stimulating electrode, 2: recording electrode, black circle: active electrode, white circle: reference electrode, (**b**) Sensory nerve action potentials. 1: affected-side distal, 2: affected-side proximal, 3: unaffected-side distal, 4: unaffected-side proximal. Delayed latency and decreased amplitude of action potentials were observed on the affected side.

In the electrophysiological diagnostics for CTS, the recommendations provided by the American Association of Neuromuscular and Electrodiagnostic Medicine (AANEM) are the most widely referenced and well-known globally [45,46,47,48]. These recommendations suggest performing sensory and motor nerve conduction studies across the carpal tunnel, and if these tests are normal, they have been shown to possess high sensitivity (80–90%) and specificity (greater than 95%). It is also recommended to use comparative testing, segmental studies, or a combination of these tests. Needle electromyography and cervical imaging studies are useful for distinguishing between cervical radiculopathy and carpal tunnel syndrome, particularly when clinical symptoms or history are atypical. Furthermore, the AANEM has published a paper on diseases that should be differentiated from cervical radiculopathy, stating that electrodiagnosis is useful when distinguishing between these similar conditions [48] (Figure 2).

There are several papers that should be referenced regarding the electrodiagnosis of cervical radiculopathy [49,50,51]. Needle electromyography (EMG) is considered useful for diagnosing radiculopathy, with moderate sensitivity but high specificity, and it serves to complement spinal imaging. When combined with nerve conduction studies, needle EMG is useful for excluding conditions that need to be differentiated from radiculopathy, such as nerve compression disorders and polyneuropathies.

These papers provide a detailed discussion of the techniques necessary to identify radiculopathy [49,50,51]. In radiculopathy, myotonic spontaneous activity and changes in neurogenic motor unit potentials (MUPs) are commonly observed abnormalities. To accurately identify radiculopathy, it is important to select the appropriate muscles. As recommended in many studies, the minimum EMG protocol should include at least five proximal and distal limb muscles. Additionally, paraspinal muscles should typically be included in the EMG, but relying on single findings for the diagnosis of radiculopathy has some limitations. Furthermore, in addition to EMG, preserving sensory nerve action potentials is crucial for localizing the lesion to the nerve root.

However, challenges remain. McGuire et al. emphasized the importance of needle EMG of the cervical paraspinal muscles in differentiating cervical radiculopathy from carpal tunnel syndrome [52]. However, to obtain accurate and reproducible results in this examination, specific anatomical locations, needle direction, needle range, assessment scores during needle insertion, and criteria for identifying abnormalities are required. Upon reviewing the literature, they concluded that there was no unified technique presented that ensured reproducible results. They highlighted the need for a standardized and useful examination technique that is both necessary and sufficient. 

Table 1 is a summary of the entire cited articles.

## 4. Discussion

Posterior neck pain is a significant clinical symptom suggesting CS, while numbness in the hands that worsens at night or with hand use is considered indicative of CTS. Although a lack of standardization in provocative test techniques across studies presents a challenge, the Phalen test appears useful for CTS screening, while the Spurling test seems appropriate for cervical spondylosis screening. When differentiating between CTS and CS, it is essential to conduct a thorough patient interview and physical examination focusing on these clinical symptoms.

Imaging studies such as MRI and ultrasound are useful for differential diagnosis; however, several concerns remain, including variations in equipment, differences among examiners, and inconsistencies in measurement techniques. Diagnosis should not rely solely on imaging studies. It is essential to standardize protocols for image analysis to ensure consistency and accuracy across studies, while also addressing morphological variations that may depend on factors such as disease severity, race, and individual physical characteristics. Ultrasound enables a multiparametric assessment of the median nerve, including morphometric and physical properties, microcirculation, and movement [41,53]. However, despite these diverse capabilities, the strongest evidence for CTS evaluation lies in ultrasound assessment of median nerve cross-sectional area and screening for structural abnormalities at the wrist. In the studies involving DTI, there is a significant issue of lacking standardization in protocols, but this issue is often overlooked [32,54,55,56]. Factors such as region of interest (ROI) selection and variations in acquisition protocols can significantly influence the interpretation of diffusion tensor metrics. Additionally, exploring the diagnostic significance in diverse disease populations can enhance its applicability and leveraging these findings to predict outcomes or identify risk factors could provide valuable insights for personalized patient care.

Electrophysiological testing is essential for the differential diagnosis of CTS and CS [57,58,59]. Beyond confirming the diagnosis, it assesses the severity of nerve dysfunction, particularly in patients being evaluated for surgery. Needle EMG studies are not obligatory, but may be needed in those with severe disease and those in whom an alternate or concomitant diagnosis is suspected [57]. In addition, it has been suggested that somatosensory evoked potentials and motor evoked potentials recording can usefully supplement clinical examination and neuroimaging findings in assessing the spinal cord injury level and severity [58]. This evaluation provides insights into the prognosis and potential outcomes of nerve function recovery following surgery. In recent years, there have been calls for higher-quality testing, highlighting the need for continuous improvement in individual technical skills.

The most critical aspect in differentiating CTS from CS is ensuring that diagnostic errors do not lead to inappropriate treatment for the patient. We must seriously consider how to prevent such errors. Research on the differentiation between CTS and CS is limited and it must be acknowledged that few studies have proven truly useful. However, keeping in mind the necessity of distinguishing between the two in daily clinical practice, we were able to obtain some information that supports the accurate diagnosis of each condition.

For CTS, the most sensitive and specific test is nerve conduction velocity testing, which compares the median distal sensory latency across the carpal tunnel with the distal sensory latency of the radial or ulnar nerves. This test should be performed when differentiation is unclear, as it is the most reliable diagnostic tool. It also holds prognostic value for surgical outcomes, with sufficient sensitivity to detect changes in treatment response. If postoperative symptom improvement is suboptimal, comparing these results with preoperative findings can help confirm whether adequate decompression has been achieved. Rigler I and colleagues reported on patients diagnosed with EMG in a longitudinal study at a single clinic. There were better treatment outcomes compared to those who were not diagnosed using EMG [60]. This suggests that obtaining a diagnosis through EMG contributes to providing high-quality treatment for both conditions. Diagnostic tests essential for guiding patients to appropriate surgical treatment should not be omitted. Data that justify surgery serve as critical evidence for patients undergoing the procedure. Most patients requiring surgery for CTS present with at least moderate severity, which should manifest as delayed NCV. Electrophysiological testing is thus crucial in providing the rationale for carpal tunnel release surgery.

In surgeries aimed at decompressing the cervical spinal cord or nerve roots, imaging evidence of compression is indispensable to support the surgical decision. Through this review, key challenges in diagnosis were identified. One major issue is that despite the high prevalence of these conditions, several review papers have emphasized the need for high-quality research. Currently, no single clinical symptom, test, or imaging modality is uniquely sufficient for diagnosis. Therefore, it is critical to apply the most accurate knowledge available, as presented in this review, and to conduct thorough examinations along with necessary tests until a confident diagnosis is reached for each patient. The challenge that emerged is that the various tests used for diagnosis must have standardized techniques in order to lead to high-quality evidence. Moving forward, it will be useful to design and conduct high-quality studies to accumulate strong evidence. Given that these are conditions with a high patient prevalence, this is considered feasible. Additionally, such reviews should be revisited every few years. Considering the high incidence of both conditions, their impact on quality of life, and the burden they place on healthcare systems, it is important to identify research priorities that should be addressed through clinical trials.

In conclusions, accurate diagnosis is essential to guide appropriate treatment and improve outcomes. No single clinical symptom, test, or imaging evaluation stands out as uniquely useful or definitive for diagnosing either CTS or CS. Clinical and provocation tests are useful for initial screening but must be combined with other methods for reliable diagnosis. Electrophysiological testing remains a cornerstone for confirming diagnosis and evaluating nerve dysfunction, while imaging techniques like MRI and ultrasound provide valuable anatomical insights but require further standardization. Therefore, this review emphasizes the importance of approaching each patient with current knowledge, conducting comprehensive examinations, and utilizing appropriate tests to achieve a confident and accurate diagnosis.

## Figures and Tables

**Table 1:** Summary of included articles.

Author	Contents
Chow CS, et al. (2005) [17]	A literature review comparing the clinical symptoms of patients with carpal tunnel syndrome and cervical spondylosis. In carpal tunnel syndrome, nighttime hand numbness and exertion-induced hand numbness occur frequently, whereas in cervical spondylosis, neck pain is more common.
Lo SF, et al. (2012) [18]	Multicenter observational study. The clinical and electrodiagnostic characteristics of CTS, CR, and the coexistence of these conditions, known as double crush syndrome (DCS), were reported. Analysis of clinical symptoms revealed that CR-alone cases showed higher rates of neck pain, upper back pain, and wrist or hand weakness compared to other groups.
Tampin B, et al. (2018) [19]	Single-center observational study. Patients with electrodiagnostically confirmed CTS and CR, and using healthy participants were researched. CR patients showed significantly reduced vibration sensation at the main pain site and decreased pressure pain sensitivity in the dermatome compared to CTS patients.
Marco B, et al. (2023) [20]	Single-center observational study. The pain distribution was widespread and significantly overlapped between patients with different levels of radiculopathy.
Padua L, et al. (2023) [21]	Review. Clinical history and physical examination are essential for screening, their diagnostic accuracy varies. Although the specificity of various clinical examination items and patient-reported questionnaires or scoring systems is high, their sensitivity is relatively low.
Verhagen AP, et al. (2023) [22]	A systematic review of the DiTA database evaluating the diagnostic accuracy of the ULTT. Test procedures and positive diagnostic criteria of the upper limb tension tests differ.
Shen P, et al. (2023) [23]	A systematic review and meta-analysis on diagnostic accuracy of the upper limb neurodynamic test with median bias (ULNT1) for cervical radiculopathy. There is a low certainty of evidence that the ULNT1 has only fair accuracy in diagnosing CR.
Núñez de Arenas-Arroyo S, et al. (2022) [24]	Meta-analysis. The HET demonstrated the best clinical performance for detecting CTS and should be considered as the first screening test during physical examinations. The most common tests, including the DT, PT, and TT, also showed good accuracy for CTS screening.
Dabbagh A, et al. (2023) [25]	A systematic review of studies on provocative tests such as the Phalen test and Tinel’s sign. They did not support the use of standalone provocative tests for the diagnosis of carpal tunnel syndrome because the studies revealed unclear and high risks of bias.
Thoomes EJ, et al. (2018) [27]	A systematic review of the diagnostic performance of physical examination tests conducted for cervical radiculopathy. The Spurling test, axial traction, and the Arm Squeeze test may increase the likelihood of CR.
Kim HJ, et al. (2013) [31]	Literature review on differential diagnosis for cervical spondylotic myelopathy. The review concluded that oblique fine-slice images obtained from three-dimensional MRI datasets provide greater consistency, enhance clinical correlation, and have the potential to improve surgical decision-making and outcomes.
Evans AG, et al. (2023) [32]	A meta-analysis focusing on studies that used the ADC for CTS. The ADC of the median nerve was lower in CTS patients compared to controls at the levels of the hamate and pisiform bones.
Rojoa D, et al. (2021) [33]	A meta-analysis focusing on studies on diffusion tensor imaging of CTS. The diffusion in the median nerve becomes more isotropic in CTS patients.
Liu C, et al. (2018) [34]	A meta-analysis to determine the optimal anatomical slice location for evaluating the median nerve using MRI in carpal tunnel syndrome. Fractional anisotropy (FA) was most suitable for diagnosis at the level of the pisiform bone, while the apparent diffusion coefficient (ADC) was more appropriate at the levels of both the pisiform and hamate bones.
Ouyang Z, et al. (2023) [35]	A meta-analysis on diffusion tensor imaging in cervical spinal cord compression. It is considered useful to combine DTI with other modalities, such as CT, to better capture the compression of nerve roots on imaging.
Meacock J, et al. (2021) [36]	Systematic review of radiological cervical foraminal grading systems. The most established systems for evaluating cervical foraminal stenosis were described by Kim and Park.
Park HJ, et al. (2013) [37]	Single-center observational study. Their new grading system for cervical foraminal stenosis based on oblique sagittal MRI provides reliable assessment and good reproducibility.
Lin TY, et al. (2022) [38]	An umbrella review on ultrasonography for the diagnosis of carpal tunnel syndrome. Sonography is a reliable tool to diagnose CTS, with inlet CSA being the most robust parameter.
Roomizadeh P, et al. (2019) [39]	A systematic review and meta-analysis on ultrasonographic assessment of CTS severity. The cross-sectional area was larger in severe cases.
Torres-Costoso A, et al. (2018) [40]	A systematic review and meta-analysis on ultrasonographic assessment of CTS. Inlet-level ultrasonography measurement appears to be an appropriate method for the diagnosis of CTS.
Yoshii Y, et al. (2020) [41]	Literature review on ultrasound diagnosis of carpal tunnel syndrome.
Chen IJ, et al. (2020) [42]	A systemic review and network meta-analysis about the diagnosis of carpal tunnel syndrome in diabetic patients. Diabetic polyneuropathy tends to result in less swollen median nerves in the CTS population.
Ikumi A, et al. (2023) [43]	Single-center observational study. In patients with unilateral symptomatic CTS, median nerve CSA correlated with BMI only on the asymptomatic side. A higher BMI is associated with a larger CSA of the median nerve.
Kim E, et al. (2015) [44]	A pilot study on ultrasonographic cross-sectional area of spinal nerve roots in cervical radiculopathy. The increased CSA of the affected nerve root compared to the unaffected side may be a useful, inexpensive, and simple supplementary clue for diagnosing cervical radiculopathy.
Werner RA, et al. (2011) [46]	Review on electrodiagnostic evaluation of carpal tunnel syndrome. This monograph addresses the various EDX techniques used to evaluate the median nerve at the wrist. It also demonstrates the limitations of EDX studies with a focus on the sensitivity and specificity of EDX testing for CTS.
Zivkovic S, et al. (2020) [47]	Quality measures in electrodiagnosis: Carpal tunnel syndrome-An AANEM Quality Measure Set
Chiou-Tan FY. et al. (2022) [48]	Review on cervical radiculopathy. Musculoskeletal mimics of cervical radiculopathy will be explored in this AANEM monograph.
Dillingham TR, et al. (2020) [49]	Review on evaluation of persons with suspected lumbosacral and cervical radiculopathy: Electrodiagnostic assessment and implications for treatment and outcomes.
Li JM, et al. (2019) [50]	Review on evaluation of persons with suspected lumbosacral and cervical radiculopathy: Electrodiagnostic assessment and implications for treatment and outcomes.
McGuire T, et al. (2024) [52]	A review of electromyography techniques of the cervical paraspinal muscles. Subsequent research will determine whether the neck muscles are helpful in the diagnosis of cervical radiculopathy. The absence of a valid reproducible cervical paraspinal technique impedes clinical and scientific understanding of cervical radiculopathy.

Abbreviations. ULTT: upper limb tension test, DT: Darkan test, PT: Phalen test, TT: Tinel test, ADC: apparent diffusion coefficient, DTI: diffusion tensor image, CSA: cervical spondylotic amyotrophy, EDX: electrodiagnosis testing, AANEM: American Association of Neuromuscular & Electrodiagnostic Medicine.
